# Pretreatment of parecoxib attenuates hepatic ischemia/reperfusion injury in rats

**DOI:** 10.1186/s12871-015-0147-0

**Published:** 2015-11-17

**Authors:** Tao Zhang, Yi Ma, Kang-Qing Xu, Wen-Qi Huang

**Affiliations:** 1 Department of Anesthesiology, the First Affiliated Hospital, Sun Yat-sen University, Guangzhou, 510080 China; 2 Organ transplantation center, the First Affiliated Hospital, Sun Yat-sen University, Guangzhou, 510080 China

**Keywords:** Hepatic ischemia/reperfusion, Parecoxib, Cyclooxygenase-2, Inflammation

## Abstract

**Background:**

Previous studies showed that cyclooxygenase(COX) was involved in ischemia/reperfusion (I/R) injuries. Parecoxib, a selective inhibitor for COX −2, has been shown to have protective properties in reducing I/R injury in the heart, kidney and brain. The aim of this study was to investigate the effects of parecoxib on hepatic I/R and to explore the underlying mechanisms.

**Methods:**

Fifty-two Sprague–Dawley rats were randomly divided into three groups: the sham-operation (Sham) group, the hepatic ischemia/reperfusion (I/R) group, and the parecoxib pretreated I/R (I/R + Pare) group. Partial warm ischemia was produced in the left and middle hepatic lobes of Sprague–Dawley rats for 60 min, followed by 6 h of reperfusion. Rats in the I/R + Pare group received parecoxib (10 mg/kg) intraperitoneally twice a day for three consecutive days prior to ischemia. Blood and tissue samples from the groups were collected 6 h after reperfusion, and a survival study was performed.

**Results:**

Pretreatment with parecoxib prior to I/R insult significantly reduced I/R-induced elevations of aminotransferases, and significantly improved the histological status of the liver. Parecoxib significantly suppressed inflammatory cascades, as demonstrated by attenuations in TNF-α and IL-6. Parecoxib significantly inhibited iNOS and nitrotyrosine expression after I/R and significantly attenuated I/R-induced apoptosis. The 7-day survival rate was increased by pre-administration of parecoxib.

**Conclusions:**

Administration of parecoxib prior to hepatic I/R attenuates hepatic injury through inhibition of inflammatory response and nitrosative stress.

## Background

Hepatic ischemia/reperfusion (I/R) is an important factor for the morbidity associated with several clinical conditions and interventions including orthotopic liver transplantation, hepatectomy, and shock. In these conditions, reperfusion induces further hepatocellular damage due to the accumulation of inflammatory cells and mediators, reactive oxygen species, and the subsequent biochemical derangements in intracellular homeostasis [1]. Hepatic I/R injury may lead to delayed graft function and a higher incidence of chronic rejections in the case of transplant recipients, or increase the complications and length of hospital stay for patients experiencing hepatectomy or shock [2].

Hepatic injuries induced by I/R are the result of complex interactions between various inflammatory mediators, among which, cyclooxygenase (COX)-derived prostanoids such as prostacyclin (PGI) and prostaglandin E (PGE) have been shown to play a critical role [3, 4]. COX is an enzyme that catalyzes the oxygenation of arachidonic acid to prostaglandin endoperoxides, which are converted into prostaglandins subsequently. There are at least two cyclooxygenase isoenzymes, COX-1 and COX-2. COX-1 is constitutively expressed in most cells and contributes to the synthesis of prostanoids, while COX-2 is undetectable in most cells in normal condition but is rapidly induced in pathological conditions, particularly in the immune system [5, 6]. Previous studies have shown that both COX-1 and COX-2 are involved in skeletal muscle and gastric I/R injuries [7, 8]. Inhibition of COX-2 by gene knock-out was found to cause a significant reduction in I/R-induced hepatic damage [9]. However, the mechanisms under the protective effect are yet to be well investigated. Thus we used parecoxib, a selective COX-2 inhibitor, which has been widely used to relieve perioperative pain, and has been indicated to have protective properties in reducing I/R injury in the heart, kidney and brain [10–12], to further gain insight into the role of COX-2 in hepatic I/R injury.

## Methods

### Experimental animals

This study was approved by the Animal Care Committee of the First Affiliated Hospital of Sun Yat-sen University, Guangzhou, China, and was performed in accordance with the committee’s guidelines for use of experimental animals. Fifty-two adult specific pathogen-free male Sprague–Dawley rats weighing between 250 and 300 g were obtained from the Animal Center of Sun Yat-sen University, Guangzhou, China. Animals were housed in individual cages in a temperature-controlled room with 12 h light–dark cycles. Food was removed 8 h before the surgery, although all animals had free access to water. The rats were randomly divided into three groups: the sham-operation (Sham) group, the hepatic ischemia/reperfusion (I/R) group, and the parecoxib pretreated I/R (I/R + Pare) group.

### Drug preparation and treatment schedule

Parecoxib (Pfizer, USA) was diluted in isotonic saline. The parecoxib treatment group was injected with parecoxib (10 mg/kg) intraperitoneally twice a day for three consecutive days prior to ischemia. The rats in sham and I/R groups were injected intraperitoneally with the same volume of isotonic saline at the same time. The parecoxib dosage was determined based on previous studies [11, 12].

### Animal model of hepatic I/R

On the day of surgery, 1 h after the last injection of parecoxib or saline, all animals were anesthetized with pentobarbital (40 mg/kg, intraperitoneal). A 3-cm midline incision was performed and the hilum of the liver was exposed. All structures in the portal triad (hepatic artery, portal vein and bile duct) to the left and median liver lobes were occluded by a clip in order to produce 70 % hepatic ischemia. The clip was removed after 60 min, and the abdomen closed. Sham-operated animals underwent midline laparotomy only, without hepatic ischemia. Core body temperature was maintained between 35.5-36.5 °C with the aid of a heating pad. Blood and liver samples of 6 rats of each group were collected 6 h after reperfusion and stored at −80 °C prior to use. The remaining rats in the I/R group and the I/R + Pare group (n = 16-18/group) were monitored for 7 days to record survival.

### Evaluation of liver injury

Blood samples were centrifuged at 4,000 rpm for 12 min to collect serum, which was then stored at −80 °C before use. Serum levels for alanine aminotransferase (ALT) and aspartate aminotransferase (AST) were determined by a serum autoanalyzer (H-7600; Hitachi Ltd., Tokyo, Japan). Liver tissue were taken from the median lobe 6 h after reperfusion and stored in 10 % formalin before being fixed in paraffin. Biopsies were then sectioned and stained with hematoxylin-eosin. Liver histologic injury was assessed using a semi-quantitative light microscopy evaluation. The histologic injury score for each sample was expressed as the sum of the individual scores for 6 different parameters: cytoplasmic color fading, vacuolization, nuclear condensation, nuclear fragmentation, nuclear fading, and erythrocyte stasis [13]. Scores for each finding ranged from 0 (0 %), to 1 (1-10 %), 2 (10-50 %), or 3 (>50 %). Each sample score was averaged over 10 microscopic fields.

### Survival study

For the survival study, the non-ischematized 30 % of the liver was resected at the onset of reperfusion. The animals in the I/R group and the I/R + Pare group were monitored for 7 days to record survival.

### Determination of TNF-α and IL-6 levels in the liver and serum

Total RNA was isolated from the liver tissue samples using the Trizol reagent (Invitrogen, Carlsbad, CA, USA) as described in the manufacturer's instructions. Quantitative PCR was performed using a Light-Cycler® 480 Real-Time PCR System (Roche, Basel, Switzerland) and the SYBR Green qPCR Master Mix (2X) (Fermentas, USA). The primer sequences: TNF-α: Forward: 5’-GTCTGTGCCTCAGCCTCTTC-3’; Reverse: 5’-CCCATTTGGGAACTTCTCCT-3’. IL-6: Forward: 5’-GCCCTTCAGGAACAGCTATG-3’; Reverse: 5’-GTCTCCTCTCCGGACTTGTG-3’. Each expression gene was normalized with GAPDH mRNA using a Delta-Delta CT method. The gene activity in the shame group was assigned as 1 as a reference. Serum TNFα and IL-6 levels were determined using an ELISA kit (Biosource International, camarillo, CA, USA).

### Hepatic myeloperoxidase measurement

100 mg liver tissue was homogenized in 1 ml of KPO4 buffer containing 0.5 % hexadecyltrimethyl-ammonium bromide by sonication and cultivated at 60 °C for 2 h. Samples were centrifuged to collect the supernatant, and then measured for protein concentration in a 96-well plate by adding samples into phosphate buffer containing o-dianisidine hydrochloride and H_2_O_2_. Light absorbance was read at 460 nm over a period of 5 minutes. MPO activity (1 unit was equal to the change in absorbance per min) was expressed as units per gram of tissue.

### TUNEL staining

Fluorescence staining was conducted using a commercially available In Situ Cell Death Detection Kit (Roche). The assay was performed according to the manufacturer’s instructions. The nucleus was stained with propidium iodide. Results were expressed as the average number of TUNEL positive cells per 10 microscopic fields.

### Measurement of iNOS and nitrotyrosine

Liver tissue samples were homogenized in lysis buffer (Promega, Madison, WI, USA) for protein extraction. The homogenates were centrifuged at 850× *g* for 10 min to collect supernatants, and then centrifuged at 10,000× *g* for an additional 10 min. The supernatants were isolated for western blot analysis. Protein concentration was determined using the BCA protein assay (Pierce, Rockford, IL, USA). Equal amounts of protein were separated on an SDS polyacrylamide gel, and then transferred onto a nitrocellulose membrane (Millipore, Temecula, CA, USA). Membranes were incubated with primary antibodies against iNOS (1:500; Santa Cruz Biotechnology, Santa Cruz, CA) or nitrotyrosine, a marker for peroxynitrite (ONOO^−^) (1:500; Upstate Cell Signaling Solutions, Lake Placid, NY). All protein bands were detected by species specific infrared fluorescent secondary antibodies (Cell Signaling Technology, Danvers, MA, USA). The relative amount of each protein was normalized by the ratio to GAPDH and analyzed using Image J (free software from the National Institutes of Health, USA).

### Statistical analysis

SPSS 16.0 was used for the statistical analysis. All data are expressed as a mean ± SE and compared by one-way analysis of variance (ANOVA) and the Student-Newman-Keuls (SNK) test. Survival rates were analyzed by the Kaplan-Meier method using a log-rank test. *P* <0.05 in two-tailed testing was considered to be statistically significant.

## Results

### Parecoxib alleviated liver tissue injury after hepatic I/R

**Fig. 1:**
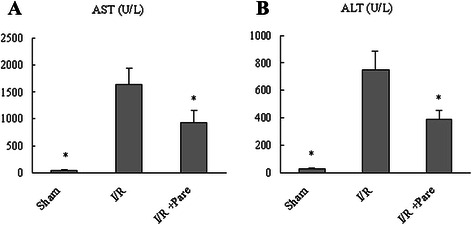
Effect of parecoxib on hepatocyte injury after hepatic I/R. Serum samples were collected 6 h after reperfusion from the sham, I/R, and I/R + Pare groups for measuring AST **a** and ALT **b**. Data presented as means ± SE (*n* = 6/group) and compared by one-way ANOVA and SNK method. *P < 0.05 vs. I/R group

**Fig. 2:**
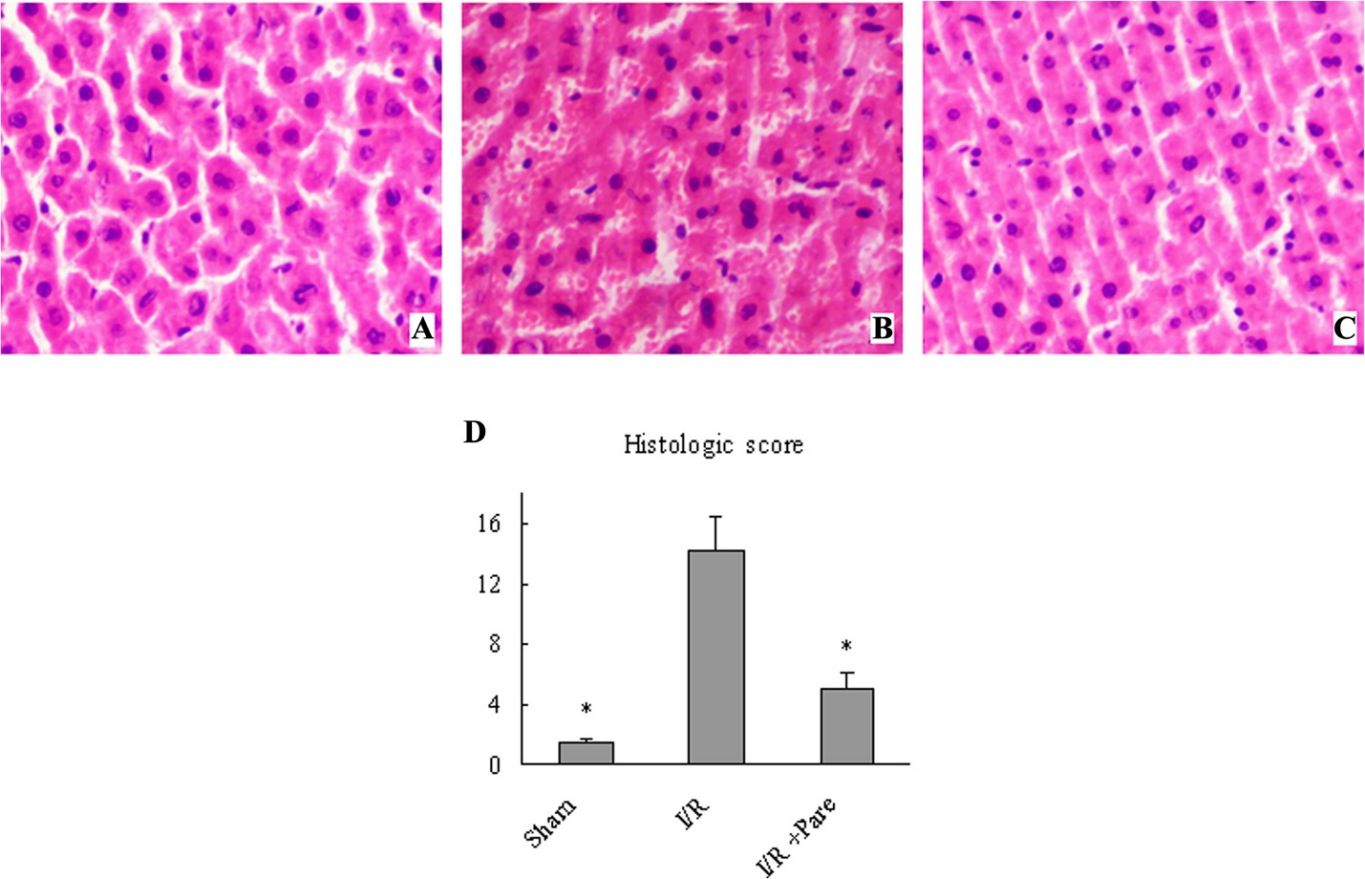
Effect of parecoxib on tissue damage and cellular architecture in the liver after hepatic I/R. Histological findings of the liver were exhibited in the sham **a**, I/R **b**, and I/R + Pare **c** groups. Liver tissues were stained with hematoxylin-eosin. Representative photomicrographs at 200× magnification. **d** Semi-quantitative histologic injury score. Data presented as means ± SE (n = 6/group) and compared by one-way ANOVA and SNK method. *P < 0.05 vs. I/R group

Hepatocyte damage was assessed by measuring serum AST and ALT levels, which increased by 46- and 28-fold, respectively, 6 h after hepatic I/R compared with the Sham group (Fig. 1). Contrastingly, treatment with parecoxib prior to I/R significantly reduced injury levels by 43 % and 48 %, respectively as compared to the control I/R group (Fig. 1). This data correlated with the alterations in tissue histological change. As compared to the sham group (Fig. 2a), livers in the I/R group showed marked coagulation necrosis, severe architerctural abnormalities and nuclear condensation (Fig. 2b), which was dramatically reduced in parecoxib-treated rats (Fig. 2c). As shown in Fig. 2d, animals undergoing I/R with control treatment exhibited a significant increase of liver histologic injury score as comapared to sham-operated animals, which was reduced by 64 % with administration of parecoxib.

### Parecoxib reduced the inflammatory cytokines in the liver after hepatic I/R

**Fig. 3:**
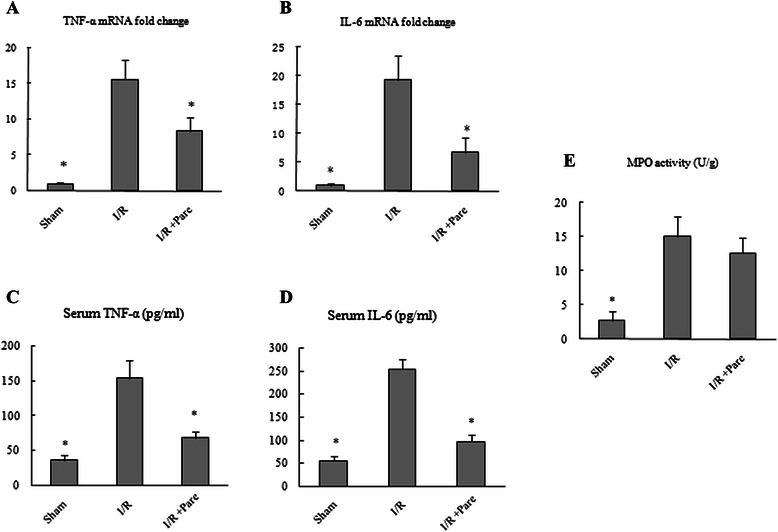
The effect of parecoxib on the proinflammatory cytokine expression and neutrophil infiltration into the liver after hepatic I/R. Liver tissues were harvested 6 h after reperfusion from the sham, I/R, and I/R + Pare groups. TNF-α **a** and IL-6 **b** mRNA expression in the liver were measured by quantitative RT-PCR analysis. Serum TNFα **c** and IL-6 **d** levels were determined using an ELISA kit. Liver tissue myeloperoxidase (MPO) activity **e**, a marker for neutrophil infiltration, was determined spectrophotometrically. Data presented as means ± SE (*n* = 6/ group) and compared by one-way ANOVA and SNK method. *P < 0.05 vs. I/R group

TNF-α and IL-6 levels in the liver and change of hepatic neutrophil infiltration were measured to ascertain the inflammatory responses after hepatic I/R. At 6 h after reperfusion, hepatic I/R resulted in a 15-fold increase of TNF-α and 18-fold increase of IL-6 mRNA expression in comparison to sham, which was decreased by 46 % and 65 % when parecoxib was administered (Fig. 3a and b). Serum TNF-α and IL-6 levels were also decreased by administration of parecoxib compared to IR group (Fig. 3c and d). When compared to the sham group, control-treated animals showed a 5-fold increase in hepatic tissue levels of MPO, which was not reduced significantly by administration of parecoxib (Fig. 3e).

### Parecoxib reduced I/R-induced apoptosis

**Fig. 4:**
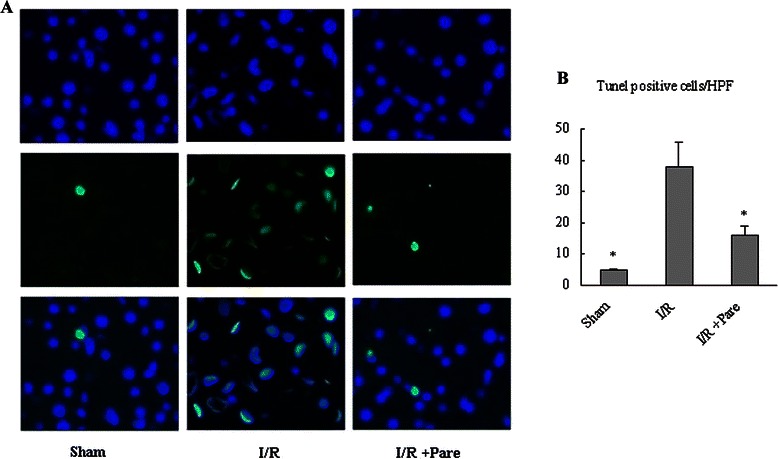
Effect of parecoxib on apoptosis in the liver after hepatic I/R. **a** TUNEL-staining 6 hours after reperfusion. **b** Results scored semi-quantitatively by averaging the number of apoptotic cells per field at 200 × magnification (*n* = 6 /group). Data presented as means ± SE and compared by one-way ANOVA and SNK method. *P < 0.05 vs. I/R group

The result of TUNEL staining showed that parecoxib decreased the frequency of apoptotic TUNEL positive cells in livers compared with IR group (Fig. 4).

### Parecoxib lowered nitrosative stress after hepatic I/R

**Fig. 5:**
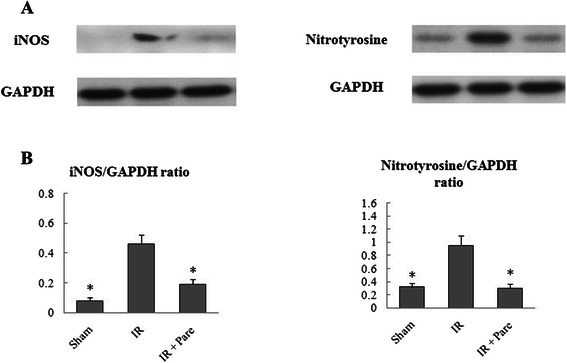
Effect of parecoxib administration on iNOS and nitrotyrosine expression in liver homogenates. **a** Western blot results for iNOS and nitrotyrosine. **b** Relative amount for iNOS and nitrotyrosine. Data presented as means ± SE (*n* = 4/ group) and compared by one-way ANOVA and SNK method. *P < 0.05 vs. I/R group

In order to determine the effect of parecoxib on nitrosative burden following I/R, hepatic tissue levels of iNOS and nitrotyrosine were evaulated. Compared with the sham group, we observed a significant increase in iNOS and nitrotyrosine protein expression in the control I/R group, which was reduced by administration of parecoxib (Fig. 5).

### Parecoxib improved survival after hepatic I/R

**Fig. 6:**
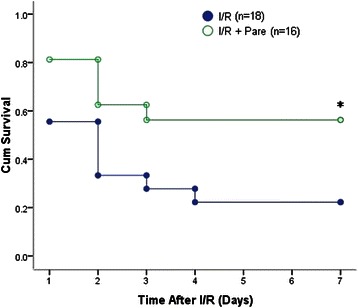
Effect of pre-administration of parecoxib on survival after hepatic I/R. Survival rates over a 7-day period following 70 % hepatic I/R were demonstrated for the I/R group (*n* = 18) and the I/R + Pare group (*n* = 16). Survival rates were analyzed by the Kaplan-Meier method using a log-rank test. *P < 0.05 vs. I/R group

Only 22.2 % of I/R animals survived 7 days after hepatic I/R. Contrastingly, parecoxib significantly improved survival rate to 56.2 % after hepatic I/R (Fig. 6) (*P* = 0.04).

## Discussion

Research aimed at the prevention and treatment of hepatic I/R has gained great attention because of the growing number of orthotopic liver transplantation, hepatectomy and other critical patients. In this study, we evaluated the protective effect of parecoxib on hepatic I/R injury. ALT and AST levels in the parecoxib-pretreated group were significantly lower compared to the control I/R group. Histological damage was milder in the parecoxib group than in the control group. Parecoxib pre-administration also reduced I/R-induced apoptosis and improved survival rate after hepatic I/R. Thus, the administration of parecoxib before ischemia appears to protect rats against hepatic I/R injury.

Ischemia reperfusion is a common pathophysiological process, which can induce altered COX-1/COX-2 expression in various organs such as in the muscle and liver [7, 14]. Studies also have shown that both COX-1 and COX-2 contribute to I/R-induced hepatic microvascular and hepatocellular injury in hepatic I/R injury [9, 15]. A nonselective COX-1/ COX-2 inhibitor, Flurbiprofen, has been shown to be protective in hepatic I/R injury [16]. In this study, we firstly elucidated the protective effect of parecoxib, a widely used selective COX-2 inhibitor during the perioperative period, on hepatic I/R injury. This further demonstrated the important role of COX in hepatic I/R injury.

Inflammatory cascades play an important role in the tissue damage during I/R. COX-2 is a major inflammatory mediator [17, 18]. Buvanendran et al. has shown that rofecoxib, a COX-2 inhibitor, is potent inhibitors of IL-6 expression through reduced prostaglandin production [19]. Feng et al. also has shown that administration of rofecoxib decreased blood levels of TNF-α and IL-6 in patients after total knee replacement [20]. TNF-α, one of the main mediators of hepatic I/R injury, is known to have deleterious effects on the hepatocytes [21]. Hyperstimulation of IL-6 has been suggested to inhibit liver regeneration [22]. Hepatic ischemia/reperfusion results in an enhanced spontaneous release of these inflammatory cytokines by Kupffer cells early after reperfusion [23]. Our study showed that parecoxib administration significantly inhibited the production of inflammatory cytokines after hepatic I/R, which may contribute to its preventive effect on the liver after I/R, although it did not reduced the level of MPO in liver tissue.

Nitrosative stress also has been recognized to contribute to the cellular damage associated with I/R injury [24]. When iNOS is upregulated in I/R injury the excessive nitric oxide (NO) produced will react with superoxide anion (O2 ^−^), creating peroxynitrite (ONOO^−^). Peroxynitrite then aggravates the injury through lipid peroxidation, apoptosis, and necrosis by nitration of tyrosine residues on tissue proteins [25]. It has been revealed that iNOS specifically binds to COX-2 and that NOS inhibition decreases prostaglandin formation [18]. Nitrotyrosine, a marker for peroxynitrite (ONOO^−^), and iNOS are often used as markers of nitrosative stress. In the present study, we observed that parecoxib administration lead to decreased iNOS and nitrotyrosine levels in hepatic I/R, which suggested that parecoxib decreased the nitrosative stress caused by I/R. This result is consistent with the decrease in number of apoptotic cells.

## Conclusion

In summary, the study showed that pretreatment of parecoxib reduced I/R-induced liver injury, inflammatory response, and nitrosative stress, and improved survival rate after hepatic I/R. These results provides evidence that parecoxib, an analgesic widely used perioperatively, could be a protective pharmacological strategy to prevent hepatic I/R injury.
